# Green Infrastructure, Ecosystem Services, and Human Health

**DOI:** 10.3390/ijerph120809768

**Published:** 2015-08-18

**Authors:** Christopher Coutts, Micah Hahn

**Affiliations:** 1 Department of Urban and Regional Planning, Center for Demography and Population Health, Florida State University, 113 Collegiate Way, Tallahassee, FL 32306, USA; 2 National Center for Atmospheric Research, Boulder, CO Postal Code 80305, USA; E-Mail: micah.hahn@gmail.com; 3 Division of Vector-Borne Diseases, Centers for Disease Control and Prevention, 3156 Rampart Road, Fort Collins, CO 80521, USA

**Keywords:** health, nature, natural environment, greenspace, green infrastructure, urban planning, built environment, ecology

## Abstract

Contemporary ecological models of health prominently feature the natural environment as fundamental to the ecosystem services that support human life, health, and well-being. The natural environment encompasses and permeates all other spheres of influence on health. Reviews of the natural environment and health literature have tended, at times intentionally, to focus on a limited subset of ecosystem services as well as health benefits stemming from the presence, and access and exposure to, green infrastructure. The sweeping influence of green infrastructure on the myriad ecosystem services essential to health has therefore often been underrepresented. This survey of the literature aims to provide a more comprehensive picture—in the form of a primer—of the many simultaneously acting health co-benefits of green infrastructure. It is hoped that a more accurately exhaustive list of benefits will not only instigate further research into the health co-benefits of green infrastructure but also promote consilience in the many fields, including public health, that must be involved in the landscape conservation necessary to protect and improve health and well-being.

## 1. Introduction

**Figure 1:**
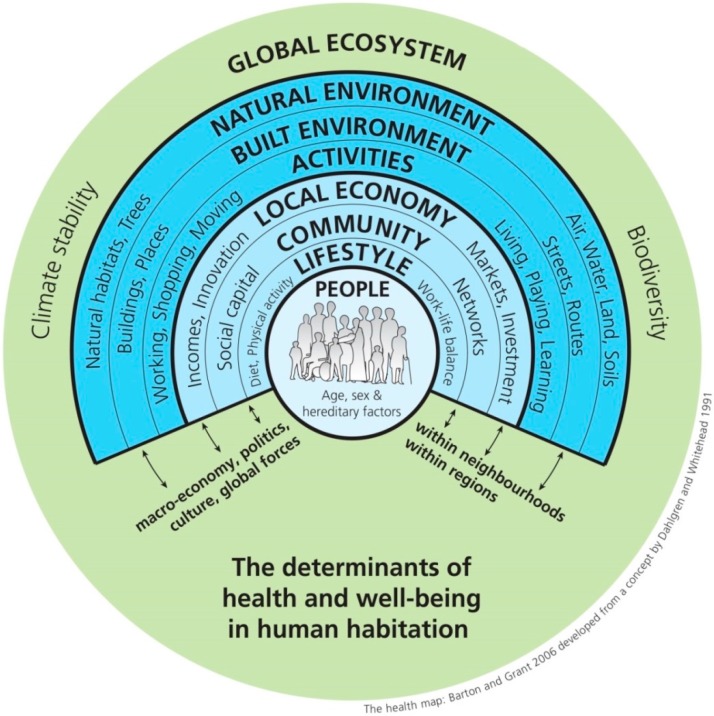
An Ecological Model of Health.

Contemporary ecological models of health have evolved to prominently feature the natural environment as fundamental to the ecosystem services that support human life and health [1]. In these models, the biosphere, landscape, and natural environment are the foundation of health and well-being (e.g., Figure 1) [2,3]. Despite this, the natural environment receives relatively little attention in health research and promotion [4]. Within the relatively small but growing body of extant nature and health research, a number of excellent studies have focused on distal determinants of health such as physical activity, social capital, and stress, but there are numerous other fundamental ways that the landscape and green infrastructure (GI) support health (e.g., infectious disease modulation, food, climate regulation). Reviews of nature and health literature have tended—granted, at times intentionally—to focus on a subset of ecosystem services or health benefits. Failing to fully represent the numerous health co-benefits of GI undersells the importance of GI, and underselling the importance of GI to health puts both GI and health at risk. The purpose of this paper is to present a more complete survey (limited by what can be included in one paper) of the array of empirically-supported human health co-benefits of GI.

**Figure 2:**
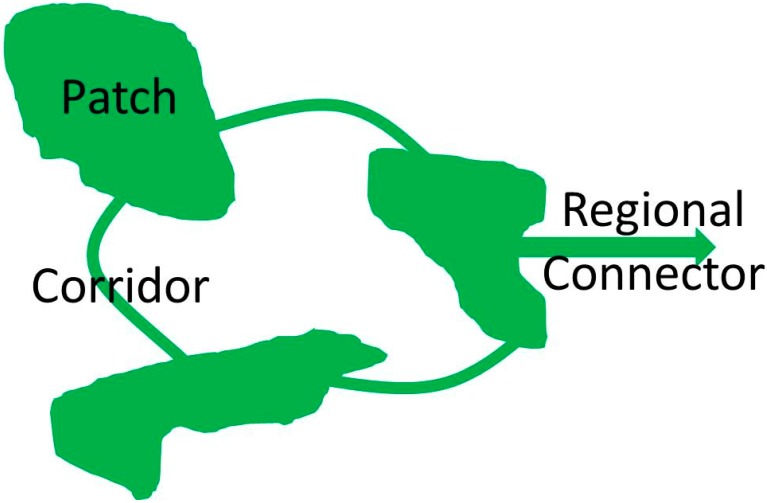
Green infrastructure.

**Figure 3:**
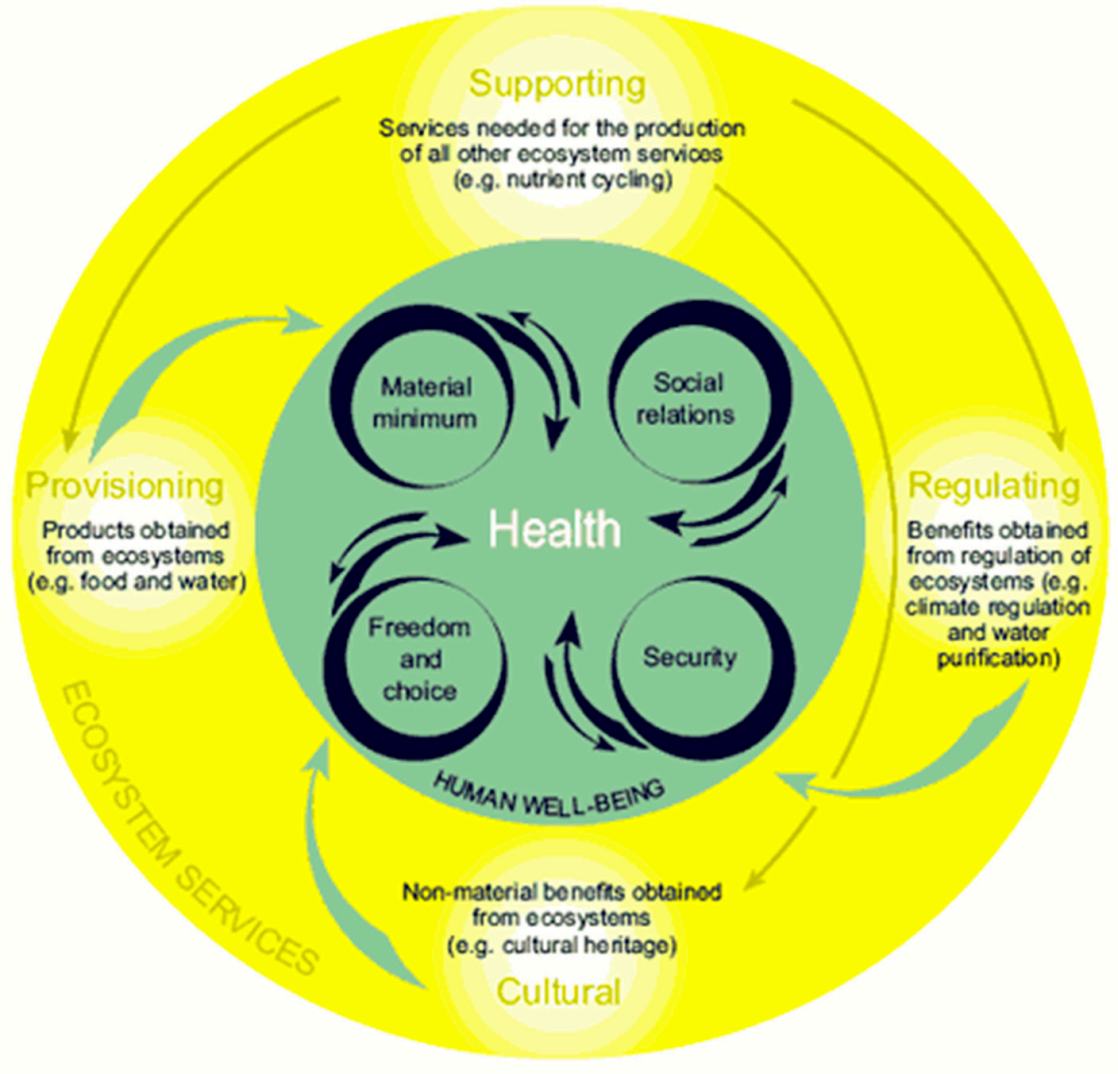
Ecosystem services and health.

The conceptual framework guiding this review is rooted in the succession of Figure 1, Figure 2 and Figure 3: The natural environment is fundamental to health (Figure 1); green infrastructure is a landscape conservation strategy that produces the structure of the natural environment necessary for ecosystem functioning (Figure 2); the ecosystem services supported by GI mediate the relationship between GI and health (Figure 3).

## 2. Green Infrastructure and Ecosystem Services

Neighborhood and national parks, parkways, forests, community gardens, and the myriad other forms of conserved private and public components of natural landscape (greenspaces), taken together and considered as a system, are what constitute a community’s green infrastructure. In urban environments, this infrastructure can include not only landscape patches and corridors but also other representations of nature (e.g., green roofs, street trees) that provide health-supporting ecosystem services without requiring the same level of consumption of finite urban land. A widely cited definition of green infrastructure is “an interconnected network of greenspace that conserves natural ecosystem values and functions and provides associated benefits to human populations” [5]. At the very heart of this definition are the benefits the natural environment provides to humans, but adopting this definition in no way discounts the environmental benefits of GI. Rather, it acknowledges that the two are complementary; environmental protection in the form of GI implementation results in human benefits. Among the highly intertwined environmental, social, and economic benefits of green infrastructure are the health benefits associated with protecting GI as our “natural life-support system” [6].

Figure 2 is an abstraction of a green infrastructure matrix comprised of interconnected patches and corridors ideally connected to larger scale matrices by regional connectors. This system of GI is essential to ecosystem functioning. In urban environments, this system of greenspace and other representations of nature are “infrastructure” as GI is intertwined within the often more visibly dominant superstructure of the built environment. The built environment is always overlaid on the regional and global superstructure of the natural environment, with GI in built environments simply being representations of the environment on which the built environment is imposed. Despite our belief that the natural environment should always be considered the superstructure on which the built environment is dependent, the term GI is used throughout this paper. This concession is made because GI supports, from within, the functions of the built environment and because it could also be considered infrastructure to the biosphere and ecosystems that have both local and global benefits to health.

An ecosystem is defined as “a biological community of interacting organisms and their physical environment” [7]. Despite the terseness of this definition, it encapsulates an extremely complex set of interactions where organisms are interacting with one another, organisms are affecting their environment, and the environment in turn is affecting the organisms that inhabit it. Among this triad of potential interactions, one holds the most pertinence for understanding the human health benefits of GI conservation: the influence of the physical environment on human organisms. Ecosystem services are the benefits that humans obtain from ecosystems, and the focus here is on the ecosystem services that GI provides to humans to sustain and enhance health and well-being.

The litany of ecosystem services can be categorized into provisioning, regulating, cultural, and supporting [8,9]. Figure 3 portrays the interdependence of these domains of service and how they are necessary for health.

The *provisioning* services are likely the first to come to mind when considering the products of nature essential for health. This includes the water produced as a service of the hydrological cycle, but also the plant and animal materials used as food and to make clothing and the natural resources used to produce energy. These services allow humans to exist. *Regulating* services are those necessary for our sustained habitation of the earth such as the purification of water as it migrates through the soil. These services also include climate regulation, carbon sequestration, flood control, biological regulation of infectious disease, and the soil fertility and pollination necessary for food production, among other services. These services are the ones most likely taken for granted by most humans—the hidden services that are essential to the continued quality and abundance of many provisioning services. The *cultural* services encompass the non-material benefits of nature. These benefits include those obtained from recreation in greenspace, the economic benefits generated from people visiting greenspaces, and the aesthetic and spiritual experience felt when observing or being immersed in the natural environment. *Supporting* services are those services that are necessary to produce all other ecosystem services. These are services such as soil formation and nutrient and water cycling on which the provisioning, regulating, and cultural services are dependent. Supporting services have also been classified as *habitat* services, which brings to the fore the overarching role of the landscape to the lifecycles of species and the biodiversity necessary to maintain resilient ecosystems [10,11,12].

The services to health are outlined in Table 1. This is not an exhaustive list of every conceivable service (e.g., there are almost certainly spiritual services), but rather a comprehensive list based on the availability of evidence connecting GI to the health benefits of these services.

**Table 1:** Categorization of ecosystem services supported by green infrastructure.

Category of Service	Ecosystem Service
Provisioning	Water quantity and quality
	Food quantity and quality
	Medicine
Regulating	Air quality
	Infectious disease modulation
	Climate regulation
Cultural	Physical activity
	Mental health
	Social capital

Some health-supporting ecosystem services come simply from the presence of GI (e.g., water, air, heat reduction), others from access (e.g., physical activity), and yet others from mere exposure to GI (e.g., stress reduction). In all instances, the focus here is on how GI supports ecosystem services. This is a step further back “upstream” away from proximate (micro/interpersonal) level influences on health in favor of a focus on the intermediate (meso/community) and foundational (macro) levels [13,14].

## 3. Water

Green infrastructure in urban and rural environments plays a vital role in the continued provision and control of the quantity and quality of the most essential of life-supporting elements: water. The role GI plays in regulating water quantity stems foremost from its role in the hydrological cycle. Green infrastructure is also important to water quantity for its ability to facilitate the recharge of groundwater stores and control surface runoff volumes. Green infrastructure supports water quality through its ability to filter pollutants that fall with the rain and also pollutants that are collected in surface runoff. 

**Figure 4:**
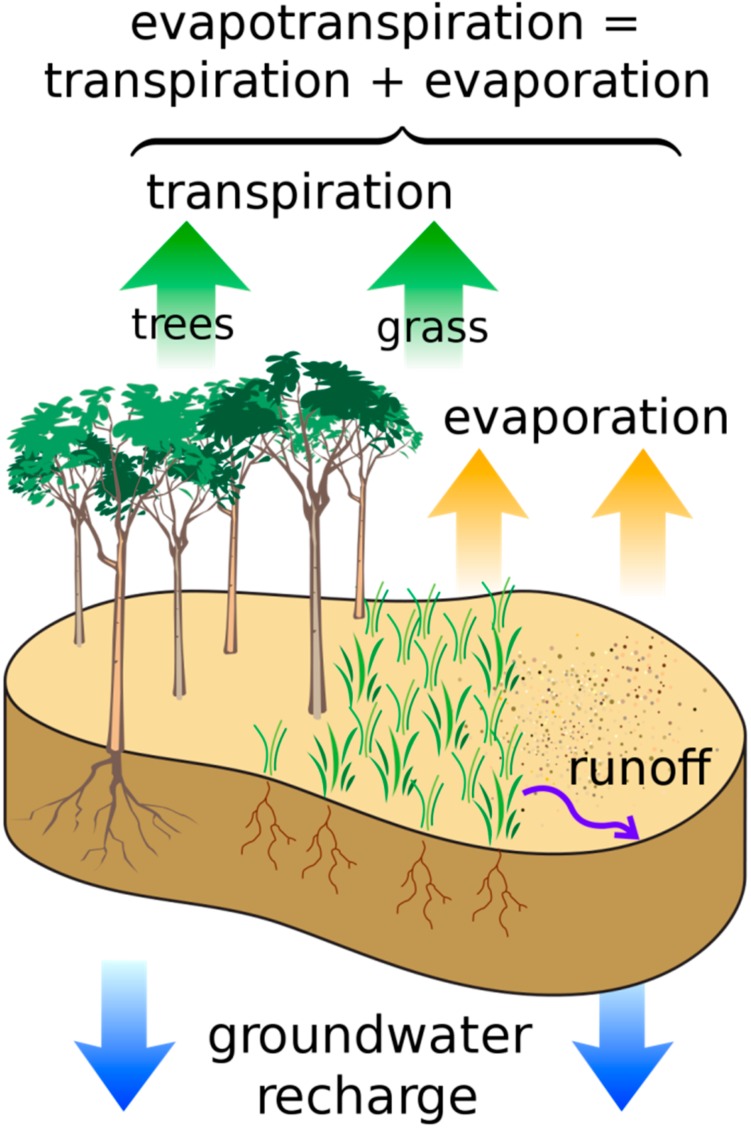
Evapotranspiration.

Green infrastructure, and forests in particular, play a key role in the hydrological cycle as it facilitates the infiltration and storage of water in soils and releases water back into the air through transpiration [15] (Figure 4). Transpiration is the process by which water drawn out of the soil is released into the air through the process of plants “breathing” [16]. Rain brings this moisture back to Earth to replenish surface water sources, and its migration through soils leads to its accumulation in groundwater reservoirs. This rainfall is also essential to sustain the GI that both released it and depends on it for its continued function in the hydrological cycle. While this may seem an elementary review of some basic science, it is likely that many people do not often contemplate their early lessons in biology and the essential role GI plays in this process essential to health and survival. Protecting the GI that supports the hydrological cycle is public health promotion at its most fundamental level.

## 4. Food

The production of food depends on three ecosystem processes in which GI plays a vital role. These are primary production, nutrient cycling, and pollination. Primary production is “the synthesis and storage of organic molecules during the growth and reproduction of photosynthetic organisms” [17]. Photosynthetic organisms, plants and some bacteria, feed themselves by capturing energy from the sun. Of all the energy that is captured by all the plants on earth, humans consume from one quarter to one half of it [18,19]. The autotrophs, such as plants, that feed themselves through photosynthesis also feed *all* other organisms on earth…eventually. As a heterotroph, humans can obtain energy only from feeding on other organisms. These organisms are autotrophs that capture energy from the sun or other heterotrophs that capture energy from feeding on other heterotrophs that somewhere down the food chain fed on autotrophs. Without the primary production of plant material, there would be no food from either plant or animal sources and human life would cease.

Pollination is another supporting ecosystem service needed for food production. The majority of the world’s crops consumed by humans are completely to moderately dependent on animal-mediated pollination, and a diminished landscape jeopardizes the ability of pollinators to do their jobs [20]. Green infrastructure provides the habitat necessary for the bees, moths, butterflies, beetles, and bats that carry pollen from male to female plants that then bear fruits and vegetables that humans consume (or that are fed to other animals that humans later consume). Green infrastructure also provides the habitat for the biological control agents that prey on crop pests [21]. (Plants themselves are used to diversify crops and repel pests. This is done by using plants to visually camouflage crops, dilute attractive stimuli, and repel pests chemically). Even modern industrial agriculture depends on pollinators to produce food such as cucumber, pear, apple, cherry, watermelon, broccoli, blueberry, almond, and many others. The annual economic value of the pollination service that bees alone provide has been estimated at $14.6 to $40 billion in the US [22,23]. Declines in certain types of pollinators providing this essential ecosystem service have been reported on every continent except Antarctica [24], and it is suspected that reduced habitat and reduced biodiversity have played roles in their demise.

## 5. Medicine

The loss of GI and biodiversity has implications for biomedicine as well as public health. As the diversity of life on earth is diminished through the loss of GI, so too is the source of many pharmaceuticals currently in use and the unmeasured potential of yet undiscovered medicines from terrestrial and marine plants, animals, and microbes. Green infrastructure provides a rich reserve of compounds that can be utilized in pharmaceuticals [25]. At least half of all prescribed drugs in the US come either directly from natural sources or are derived from natural sources [26], and “thirty percent of the drugs sold worldwide contain compounds derived from plant material” [27].

There has been a swelling of research investigating the health benefits of bioactive compounds [28]. Polyphenols, the most abundant antioxidant in the human diet, have preventive properties for degenerative diseases such as cardiovascular disease and cancer [29]. Phytoestrogens, found most notably in soy, have been associated with lowered risk of osteoporosis, heart disease, breast cancer, and menopausal symptoms [30]. Without the conservation of the GI that supports biodiversity, many bioactive compounds and their potential health benefits could be lost.

## 6. Air

Selected forms of vegetation, most notably trees, have the capacity to capture both gaseous and particulate airborne pollutants [31,32]. Gases are removed from the air via uptake by leaf stomata (pores on leaves), absorption through leaf surfaces, and adsorption (or adherence) to plant surfaces. Particulate matter removal occurs through deposition on leaves and other plant surfaces. Particulate matter is held on these surfaces by either being stuck on impact or through an adsorptive chemical process [33]. (Plants can remove airborne pollutants through the various chemical reactions that occur on plant surfaces often with the aid of precipitation. The process by which trees remove pollutants from the air without the aid of precipitation is called dry deposition).

Trees remove tons of air pollution annually. In the southern US cities of Houston and Atlanta, both with similar tree coverage, annual removal of particulates by trees was 4.7 and 3.2 tons per square mile respectively [34]. In fact, urban trees alone in the US remove 711,000 metric tons (1,567,486,684 pounds) of pollutants annually estimated at a $3.8 billion value [35]. Intertwined in the externality value of trees could be perceived health benefits, but this is not explicit. A more recent study explicitly focused on health benefits found the avoidance of human mortality and acute respiratory symptoms attributed to the uptake of air pollution by trees and forests in the US was $6.8 billion in 2010 [36]. In other cities struggling with air pollution issues such as Beijing, trees removed 1261.4 tons of pollutants from the air in 2002 [37]. Urban trees in other rapidly expanding Chinese cities have been found to be important to the ecosystem service of air pollution abatement [38]. 

A handful of studies have tied the ability of trees to capture pollutants to human health outcomes. In New York City neighborhoods, Lovasi *et al.* found an association between tree density and a lower prevalence of early childhood asthma [39], but Lovasi, *et al.* have since determined the validity of these findings may have been compromised by the scale of their analysis and the limited tree type examined [40]. Employing a natural experiment study design, Donovan *et al.* [41] examined the natural destruction of trees by the emerald ash borer across massive swaths of the eastern US to discern if the loss of trees had any effect on cardiovascular and lower respiratory mortality. They were able to reveal a strong relationship between many more thousands of deaths from these two causes and the destruction of trees by the emerald ash borer. A number of ecosystem services presented in this paper could be potential mechanisms that contribute to this relationship, but it is suspected that air pollution abatement is one such mechanism, especially considering the outcome of lower respiratory mortality.

While there is mounting empirical evidence to support Frederick Law Olmstead’s century old claim that parks and trees are the “lungs of the city,” GI is not a stand-alone solution for cleaning the air for an expanding global population. GI filters a number of harmful pollutants from the air (ozone, nitrogen oxides, sulfur dioxide, particulate matter, and carbon monoxide) but at nowhere near the levels that would counteract the rate of emission of pollutants into the atmosphere. GI is not a substitute for reducing pollution at its source. There is also a point where levels of air pollution can harm the very GI that is filtering these pollutants and providing other ecosystem services.

This section on the air quality benefits of GI is a good place to confront the fact that GI not only produces benefits to health but also can pose threats to health and well-being [42]. Using the example of air quality, GI can remove pollutants from the air, but trees, and particularly grasses, also produce and release pollen that can cause mild to potentially debilitating allergic reactions. With the prevalence of allergic diseases on the rise worldwide [43], this is a legitimate cause for concern. Green infrastructure can contribute to an increased prevalence of allergic reactions if the type of GI introduced is not carefully considered. This can be at least partially remedied by protecting (and often reintroducing) native GI species. Many non-native ornamental plants and trees installed as GI are highly allergenic and cause more harm to health than their naturally occurring counterparts [44]. 

## 7. Infectious Disease Modulation

Infectious disease ecology is a rapidly evolving field focused on understanding how hosts, pathogens, vectors, and their environment evolve, respond, and interact with one another in ways that influence the spread of disease [45]. The landscape and GI are increasingly recognized as a barriers or conduits of disease amplification and spread in human, domestic animal, and wildlife populations. Disease ecology and its complementary fields all appreciate that the consequences of altering GI is part of a dynamic process of feedbacks and cascading impacts of ecosystem perturbation that may not be evident for several years [46].

A number of studies from these fields have demonstrated the various pathways through which patterns of GI can mediate infectious disease spread [47]. Green infrastructure can influence disease risk directly, for example, through habitat availability for vector and zoonotic reservoir populations. Alternatively, impacts may be more indirect—by shifting the biodiversity of an ecosystem in ways that limit or propagate pathogen spread within the reservoir community.

### 7.1. Zoonotic Disease

Nearly two-thirds of human infections are zoonotic [48], meaning they are animal pathogens that have been transmitted to humans. The long-term host of a pathogen is referred to as the disease reservoir. Studies from a variety of ecological contexts have documented how landscape changes can influence the frequency and intimacy of interaction between humans and zoonotic disease reservoirs and propagate pathogen transmission between species. 

One example of these interactions is the trade in wild animal meat. Hunting and sale of wild animals, or bushmeat, is an important source of income and protein for many rural communities in the tropics, with estimates exceeding five million tons of meat extracted annually [49]. Intimate blood and bodily fluid contact between the hunters and the hunted creates an effective interface for the introduction of novel, zoonotic infectious agents into the human population [50].

In addition to increasing the frequency of direct contact between humans and zoonotic disease reservoirs, the management and alteration of landscape can influence indirect interactions among people, wildlife, and domestic animals through utilization of shared resources. In Kibale, Uganda, a long-term ecological study has documented the presence of forest fragments leftover on land unfit for agriculture near the border of Kibale National Park that are areas of intense human-primate interaction [51]. The result of these overlapping living spaces and resources is increased bacterial transmission among species as evidenced by the genetic similarity of gut bacteria in primates, humans and livestock living near the same forest fragment [52]. Moreover, the degree of genetic similarity of these bacteria parallels the relative degree of anthropogenic disturbance in the fragments (based on measures of encroachment, forest clearing rates, and intensity of human use) [52].

### 7.2. Vector-Borne Disease

Vector-borne diseases are transmitted to humans by arthropods, such as mosquitoes, ticks, fleas, and other blood-sucking insects. Almost 30% of emerging infections in the last decade were vector-borne diseases and this number has risen since the 1940s [53]. Changes to the landscape can affect vector breeding sites and the microclimate in ways that significantly affect the rate of larval development, biting frequency, and survival of the vector [54,55]. 

The global demand for cropland and pasture has driven the expansion of agricultural land in the last half of the 20th century, primarily at the expense of intact tropical forests [56]. The impact of deforestation in the tropics on vector-borne disease risk has been shown across continents using fine-scale field studies as well as more coarse-scale remote sensing studies used to assess large geographic extents [57]. For example, in western Kenya, Afrane *et al.* [58] showed that the average ambient temperature in deforested areas was 0.5 °C warmer than forested areas. As a result, the reproductive cycle of *Anopheles* mosquitoes living in the deforested areas was accelerated by almost three days, resulting in an increased biting frequency and risk of malaria transmission to humans.

## 8. Climate Regulation

Climatic norms are changing, and expert opinion largely concurs that the magnitude of the health risks in all countries and regions will also change, perhaps in unexpected ways [59]. As climatic conditions continue to change, GI will change, and so too will the ecosystem services on which health depends. Our nascent understanding of how global health already has been, and will continue to be, affected by climate change reveals that GI, yet again, plays a critical part in improving the human condition [60]. Green infrastructure plays an important role in both mitigating climate change and also adapting to changing climatic conditions and events, and any assessment of how climate change will affect health must include the alterations that humans make to the landscape [61].

There has been a small surge in the literature in recent years exploring the potential health impacts of global climate variability. Although growing, guidance on this subject is still sparse. The health outcomes that have been studied have largely been limited to those associated with heat waves and air pollution [62] with a steadily growing literature in vector-borne and infectious disease ecology. There is a recognition that there are a number of other environmental indicators (e.g., use of renewable energy) that are needed alongside more traditional indicators to measure the health effects of climate change [63]. Table 2 provides a synopsis of the climate change and variability events and their corresponding health effects.

**Table 2:** The Health Effects of Climate Change.

Climatic Event	Intermediary	Health Outcome
Heat waves	direct to →	Heat stress, stroke
	Increased ground-level ozone, pollen	Respiratory disease exacerbation
Increased mean temperature	direct to →	Positive: Less hypothermia
	More hospitable to disease vectors (e.g., mosquito, ticks)	Vector-borne diseases (e.g., Lyme, malaria, dengue)
	More hospitable to infectious disease agents (e.g., bacteria)	Food-poisoning, infectious disease (e.g., cholera)
Ozone depletion	UV radiation	Skin and eye maladies
Drought	Water/food shortage	Dehydration, malnutrition
	Lack of water safety	Water-borne disease
Extreme weather event (e.g., flooding, tornado, hurricane)	direct to →	Injuries, drowning
	Population movement	Conflicts
	Lack of food/water safety	Water-borne disease, malnutrition
Sea-level rise	direct to →	Injuries, drowning
	Population movement	Conflicts
	Water/soil salinization	Dehydration, malnutrition
Climate change generally	Stress	Mental health

*Note:* Adapted from [64]. Compiled with data from [59,65,66,67].

Of course, projecting the level of increased morbidity and mortality caused by climate change is extremely complex and fraught with uncertainty. Nonetheless, there have been some efforts by public health researchers to quantify the increased health risks attributable to the conditions that may result from a changing climate. It has been shown that climate change has already affected human health and that the associated risks from a number of conditions will likely increase over time ([68], Table 7.2). In the year 2000 alone, climate variability was likely responsible for over 150,000 deaths worldwide with almost 90% of this increase in the health risks falling upon children [69]. While the methods used to achieve this estimate have received some criticism [70], the evidence we have demonstrates that climate change is already having an effect on health and that this effect is likely to become more prominent.

### 8.1. Infectious Disease and Climate

The ecology of many infectious diseases is tightly linked to climate via impacts on the life cycle of pathogens, the arthropod vectors that transmit them, and the animal reservoirs that host them [71]. Changes in climate have already had significant impacts on many infectious diseases [72,73,74], and an increasing number of studies are trying to predict future climatic impacts on infectious disease risk [71]. There are three primary mechanisms through which climate change can affect vector-borne and zoonotic diseases: (1) geographic range shifts of vectors or reservoirs; (2) changes in rates of development, survival, and reproduction of vectors, reservoirs, and the pathogens that they carry; and (3) changes in biting rates of infected vectors or the prevalence of infection in reservoir or vector populations, which affects the likelihood of transmission resulting from contact with a human [55,72,75].

There are numerous examples of the impacts of climate on the development, survival, and reproduction of vectors, reservoirs, and the pathogens they carry. For example, higher temperatures have been associated with increased tick nymphal activity as a result of faster egg development after the birthing season [76]. Mosquito populations are affected by precipitation, particularly during breeding, although the direction of the effect is dependent on the breeding requirements of each mosquito species, some of which prefer to breed in small pools while others prefer edges of larger, more stable water bodies [73]. Mammalian pathogen reservoirs can be affected by high temperature directly through heat-related mortality as has occurred in the flying fox (*Pteropus* spp.) population in Australia [77], or indirectly through increases in vegetative habitat and food resources related to increased temperature and precipitation [78]. Many studies have shown that the extrinsic incubation period—the interval between the acquisition of an infectious agent by a vector and the time when a vector can transmit the infection—is highly temperature dependent [79,80,81,82]. As the temperature increases, the rate of pathogen development inside the vector increases so that vectors can transmit the infection more quickly after a blood meal.

### 8.2. Green Infrastructure and Carbon Sequestration: Mitigation and Primary Prevention

Green infrastructure, and in particular trees, can mitigate the potential adverse health effects caused by climate change through the ability of plants to capture and store carbon from the greenhouse gas CO_2_. In 2011, CO_2_ accounted for 84% of the total greenhouse gas emissions in the US [83], the greatest source of these emissions coming from the combustion of petroleum, coal, and natural gas.

The change in climate that will occur over the next 30 to 40 years has been determined by the level of carbon dioxide already in the atmosphere [84]. Even if all emissions were to stop today, we would be dealing with the ramifications of current concentrations of carbon in the atmosphere for at least the next 40 years and possibly much longer. While it is absolutely necessary to reduce emissions to prepare for life beyond 40+ years, it is also advantageous to health to capture and sequester the carbon already in the atmosphere. A study of 10 major US cities found the gross carbon sequestration rate of urban trees to be 22.8 metric tons of carbon/year (a $460 million/year value) [85]. In the US in 2005, the net carbon sequestration of the forest sector offset 10% of CO_2_ emissions [86]. 

Larger and more mature trees store more carbon than younger trees, but this does not necessarily mean that pristine, old-growth forests are the most efficient at capturing carbon. In central California, observed rates of annual carbon sequestration ranged from 35 pounds for small trees to 800 pounds for large, mature trees [34], but younger trees in rapid growth stages capture carbon at a higher rate than their more mature and slower-growing counterparts. Also, in mature forests, carbon sequestration is counterbalanced by the decomposition and release of carbon from trees that have reached the end of their lifespan. The selective harvesting of trees for wood products keeps carbon stored in the dry wood (where it makes up half of the material mass) for a longer period as compared to if trees were allowed to decay, but the tree residues and roots left behind to decay after harvesting may offset gains in the dry wood storage of carbon. Harvested wood burned for fuel releases sequestered carbon back into the atmosphere immediately, but burning wood for fuel is a much more carbon neutral option as compared to fossil fuel combustion. Indeed, it is the carbon released from fossil fuel combustion that we are counting on trees to capture. The ability of forests to act as net carbon sinks is complicated by changing climatic conditions, soil fertility, varied carbon sequestration rates of tree species, and a range of other issues that are not completely understood [87]. The lesson here is that when considering the potential of trees and forests to act as carbon sinks, the sustainable harvesting of forest products for fuel and building materials may not be contrary to efficient carbon sequestration, climate change mitigation, and health promotion.

### 8.3. Green Infrastructure and Extreme Weather and Climatic Events: Adaptation and Secondary Prevention

The ability of GI to sequester carbon will aid in mitigating the threats from impending climate change, but change is already underway, and adaptation is therefore a necessary complement to mitigation.

The global migration to cities combined with the increased frequency of extreme weather events is a cause for concern. The destruction of coastal mangroves, forests, wetlands, coral reefs, and vegetated dunes has serious consequences for the 40% of humanity who reside within 100 km of the ocean shorelines and at less than 50 m above sea level. These natural features can diminish the flooding, storm surge, and landslide activity that occurs during extreme storms [88]. Noteworthy is the fact that the protection coastal vegetation (such as mangroves) provides against potentially destructive wave action is not linear but rather increases exponentially the more area that is conserved [89].

Heat is also another climatic condition that threatens health and well-being. There is no question that the globe is warming. The first 13 years of the 21st century have accounted for 13 of the 14 warmest years of global average surface temperatures on record (since 1850) with each of the last three decades warmer than the previous decade culminating in 2001–2010 as the warmest decade to date [90]. This is particularly pronounced in cities.

First recorded in 1833, the phenomenon of cities consistently having higher temperatures as compared to the surrounding countryside has now been demonstrated beyond a doubt [91,92]. The *urban heat island* effect remains the most intensively studied climatic feature of cities and a major focus among a much wider field of urban climatology [93,94]. The US Environmental Protection Agency reports that “the annual mean air temperature of a city with 1 million people or more can be 1–3 °C (1.8–5.4 °F) warmer than the surrounding region” [95]. Other estimates report a temperature difference as high as 7 °C (~12 °F) [96]. As ambient temperatures rise, so too will the risk of the direct and indirect adverse effects heat has on physiological processes. Some direct effects are less severe such as heat cramps, heat edema (swelling), heat syncope (fainting), while others such as heat exhaustion and heat stroke can cause organ damage and death [97,98].

When temperatures are high in cities, this increases the demand for the electricity to cool buildings, which creates more greenhouse gases that trap heat. It does not require large changes in temperature to translate into large increases in demand for energy. For every 0.6 °C (1 °F) increase in summertime temperature, peak utility loads in medium and large cities increase by an estimated 1.5%–2.0% [99]. So, if urban areas are projected to be approximately 5 °F hotter (a conservative estimate), this could translate into the heat island being responsible for 10% of the peak energy demand [100,101]. Effective use of green infrastructure in cities as an adaptive strategy is likely to have a pronounced impact on urban environments. Green infrastructure in the form of shade trees can create a seasonal (~3 months in temperate zones) cooling energy savings of 30% [102]. Undoubtedly, a 30% reduction is substantial in the reduction in emissions that exacerbate ambient heat.

Contributing to the urban heat island is the relative lack of greenspace (and water features) in cities [91,92,103]. Greenspace can prevent the absorption of radiation by surfaces and the release of pollutants as well as cool the air through evapotranspiration. Parks within a city can have a significant cooling effect on local temperatures [104] although park size needs to exceed one hectare for significant cooling benefits with at least 10 ha needed to achieve a 1 °C reduction in air temperature (Kuttler, 1993 in German as cited by [15]). The park size needed to achieve these temperature reduction benefits may not always be achievable in urban environments, but other forms of GI also provide the co-benefit of temperature reduction. Adding as little as 10% of greenspace in the form of trees in high-density development can reduce local surface temperatures by 1.4 °C on average [105,106]. These reductions in temperature, although seemingly small, would nearly negate minor heat islands. It would also essentially negate projected local temperature increases for the next 65 years [92]. Shade also directly protects individuals from UV radiation. An individual tree can provide a Sun Protection Factor (SPF) of 6 to 10, a level of exposure to UV radiation one sixth to one-tenth of full sun [NUFU, 1999 as cited by 15]. This is important for not only preventing health issues associated with heat but also other associated health problems [107] such as skin cancer.

## 9. Physical Activity

There has been nothing less than a small explosion in the volume of literature over the past two decades examining the ecological influence of the physical environment on physical activity behavior. This movement was spurred by a recognition of the understudied influence of the physical environment in facilitating or deterring physical activity. This body of literature has largely focused on the elements of the built urban environment such as land uses, block lengths, and road patterns. A subset of this literature has examined the role of greenspaces in facilitating activity. While our understanding of the relationship between the built environment and activity has grown, a systematic review of the literature on the built environment and physical activity concluded that more studies are greatly needed [108]. Green infrastructure has been posited as a potential environmental support that can encourage the myriad health benefits gained through regular physical activity.

The majority of studies of GI and physical activity support a relationship between greener environments and higher levels of physical activity (77.5% positive or mixed) [109]. The overall conclusion is that “…the value of greenspaces as places to exercise is unquestionable,” but this assertion is qualified with an acknowledgement of the mixed findings related to GI characteristics such as accessibility [110]. The aesthetic qualities of GI influence its use for physical activity, but these characteristics are important only after access is achieved. Creating greater access requires not only a greater abundance of GI but also a more even distribution of GI.

One of the factors driving the surge in research into and advocacy for environments conducive to physical activity is the recognized need to stem the epidemic of obesity. Over one-third of adults and nearly 20% of children in the US are obese [111]. Worldwide, obesity has nearly doubled since 1980 [112]. Most of the research and initiatives aimed at addressing this epidemic operate under the assumption that more physical activity will lead to reduced obesity and its associated diseases [113]. The use of GI for regular physical activity has the potential to tip the caloric intake/expenditure equation in favor of caloric expenditure. Diet is also an absolutely, if not more [114], critical part of this equation, but the litany of other health benefits of regular physical activity should caution against focusing on diet alone.

In a review of the literature on greenspace and obesity research, Lachowycz and Jones [115] found that increased access to greenspace generally lowers the likelihood of obesity (e.g., [116], but that the cumulative findings were too inconsistent to draw any firm conclusions. Many studies have correlated measures of the physical environment with obesity based on the assumption that increased greenspace increases physical activity, which decreases obesity. For example, Ellaway *et al.* [117] showed that higher levels of neighborhood greenery were associated with both more physical activity and reduced levels of self-reported overweight and obesity. Although these types of studies are a valuable starting point, there is much work to be done to better understand the characteristics of GI that support physical activity and who is benefitting. 

Looking at a characteristic of the built environment, GI, and obesity together was an examination of how residential density and the amount of greenspace influences children’s body mass index (BMI) [118]. Population and residential density have been used as proxies to describe built environments that support physical activity—the belief being that areas with greater density are more likely to include the physical features (e.g., reduced proximities, land use mixture, more connected street networks) that support walking and biking. This was not found to be the case for adults in a study of residential density and the levels of walking for physical activity necessary to provide health benefits and reduce obesity [119], although the cross-sectional nature of this study, and others, always introduces the possibility of residential self-selection and type 1, and, in this case, a possible type 2 error. Returning to the study of children, residential density, again, was shown to have no effect on children’s BMI, but the greenness of neighborhoods was associated with lower BMI regardless of residential density. Furthermore, more greenness reduced the odds of children increasing their BMI over a two year period. As the authors note, the mechanism that may explain this is that children take advantage of more types of GI (e.g., yards, parks, vacant lots) than adults. Getting kids more active and reducing childhood overweight and obesity is critical not only to childhood health but also to physical activity behavior later in life. More active children leads to more active adults [120].

## 10. Mental Health

Exposure to GI also supports mental health, “…a state in which a person is most fulfilled, can make sense of their surroundings, feel in control, can cope with every day demands and has purpose in life” [121]. Fully achieving health requires striving for complete physical *and* mental well-being, the two being inseparable. There are a number of ways that exposure to and affiliation with the natural environment has been shown to support mental health. These include nature’s ability to reduce stress, create positive affective states, and improve cognitive functioning. 

What most often comes to the fore as the empirical basis for why GI is good for mental health is the many decades of environmental psychology and environment-behavior research examining the mentally restorative potential of exposure to the natural environment and elements of nature. The innate human preference for many elements of the natural environment and natural processes endows the natural environment with the unique ability to restore and renew “diminished functional resources and capabilities” [122], not only permitting mental restoration but promoting it so that daily physical, psychological, and social demands can be met [123]. Restoration has often been examined through the lens of either the psychoevolutionary stress recovery theory or attention restoration theory, but these two theories are not necessarily at odds and may even complement one another [124]. They “…occur alone in some circumstances, but in other circumstances they may have some form of reciprocal relationship or otherwise coincide” [125].

Recent longitudinal evidence has revealed that GI in one’s environment is indeed important in delivering sustained gains in general mental health [126]. In a rigorous study that examined the relationship between neighborhood greenness and mental health, it was found that the greenness of one’s environment was a significant independent predictor of improved mental health even when accounting for physical activity (recreational walking) and social cohesion (discussed in an upcoming section) [127].

### 10.1. Stress

Ulrich’s [128] study of hospital patients’ window views of nature has been cited almost without fail as evidence of the connection between exposure to nature and improved health. This study into the therapeutic properties of nature views revealed that hospital patients recovering from surgery had shorter hospital stays, lower intake of potent narcotic pain drugs, and more favorable evaluations by nurses if their hospital room windows allowed views of trees rather than views of a brick wall. Years later, Ulrich *et al.* targeted stress recovery more directly and focused on passive exposure to nature not through a window but by comparing the effect of viewing videotaped nature views and videotaped urban views largely devoid of natural elements [129]. It was the first such study to test the psychophysiological stress recovery theory using a number of objective physiological indicators (and affective state to be discussed in the following Affect section). Using muscle tension, skin conductance, and pulse transit time which correlates with systolic blood pressure, it was found that “…recovery from stress was much faster and more complete when subjects were exposed to the natural settings…” as opposed to urban settings [129].

A number of cross-sectional studies have shown that when exposure to nature is achieved through access, and not just viewing images or having a window view, increased access is accompanied by reduced levels of stress. Those who report visiting greenspace more frequently and spending more time in greenspaces report fewer stress-related illnesses [130], and those who do not report being stressed are much more likely to visit greenspaces at least a few days a week [131]. Although these studies do not prove causality between access to greenspace and reduced stress, individuals in the study cite “reducing stress and relaxing” and “obtaining peace and quiet without noise” as motivations for using greenspace. 

The amount of greenspace one can access has also been shown to reduce stress and improve overall health. The impact of stressful life events, the number of health complaints, and perceived general health are significantly moderated by amount of greenspace within a 3-km radius of home; those with a greater amount of greenspace report being less affected by a stressful life event than those with less greenspace [132]. More greenspace appears to be beneficial to reducing stress, but there also appears to be such a thing as too much “greenness.” An experimental study of the effect of street tree density on stress recovery revealed that there are diminishing returns with high levels of tree density [133]. Varying the level of street tree density had no significant effect on stress recovery in women. Men did experience stress recovery benefits with exposure (in videos) to moderate levels of street tree density, but these benefits diminished as street tree density increased. Results such as this speak to the importance of GI design and should be encouraging to those trying to strike the right balance between the built and natural environments. Unadulterated views of nature are not necessary to deliver stress recovery benefits. A balance between the built and natural environment may actually optimal for the delivery of some mental health benefits. 

### 10.2. Affect

Affect is “…any state that represents how an object or situation impacts a person” [134]. Affective state is represented in innate human feelings such as fear, anger, and joy which are then filtered through our learned cultural norms and expressed in emotions [135] such as anxiety, depression, aggression, and happiness. Essentially, to be human is to have feelings, which make us aware of our biologically determined affect, but how these feelings are expressed in emotions is determined by biological factors and by social learning. The focus here is not on how biological and social factors influence the expression of emotions but rather on how GI can influence emotional states that are associated with morbidity (e.g., depression).

Before Ulrich’s focus on stress, one of his first studies into the psychological benefits of exposure to nature found that viewing nature scenes brought about an increase in positive affect as measured by affection, friendliness, playfulness, and elation [136]. Similar to stress research—and at times folded into the same studies—the effect of greenspace on emotions and mood has been measured by comparing exposure to natural environments *versus* more urban and artifact-dominated environments. After viewing distressing images, Ulrich *et al.* found that subjects that viewed natural scenes reported less fear, anger/aggression, and much higher positive affect. This “recovery associated with the natural exposures was so pronounced in terms of the Fear, and especially the Anger/Aggression [sic] and Positive affects factors, that post-recovery affective states were somewhat more positively-toned than those reported during the base-line period” [129]. Not only did viewing images of nature produce affective recovery after being distressed, but subjects were better off than baseline in terms of affect.

Although controlled simulations that account for potential confounding variables have consistently revealed more positive emotional self-reports in higher levels of overall happiness and reduced anger and aggression [137], simulations cannot account for the myriad other senses, in addition to the visual, that may have an influence on one’s interpretation and response to the environment. Complementing controlled simulations are field studies that engage all the senses. In one such field experiment, persons first completed tasks that demanded focused attention and then took a walk in either natural and urban environments [125]. While no improvement was found in overall happiness when walking in the natural environment, there was an increase in overall positive affect and anger/aggression decreased relative to the pretest. The opposite pattern occurred in the urban environment. The same result of higher positive affect and happiness and lower anger and aggression was evident in an earlier field study that compared the effects of walking in natural *versus* urban environments [138].

The most recent work measuring differential affective responses to natural *versus* urban environments has employed Electroencephalography (EEG) and EEG-based emotional recognition software to measure changes in emotions while navigating city environments on a walk. It was found that pedestrians that were walking on a busy urban shopping street and then entered greenspace experienced reductions in arousal (long-term excitement) and frustration [139].

The green outdoor environments that have been found to be associated with reduced BMI in children (cited in Physical Activity section) may also provide the co-benefit of reduced aggression. It was found that less bullying occurs in children’s play spaces with a highly interactive and an engaging natural environment [140]. This finding does not allow us to conclude that the engagement with the natural environment causes less bullying, but, similar to other emerging research topics, it provides a basis from which to further explore the ability of the natural environment to instill more positive emotions in children.

### 10.3. Cognition and Attention

An enduring definition of cognition is all processes by which “…sensory input is transformed, reduced, elaborated, stored, recovered, and used” [141]. Essentially, cognition is what most would consider thinking or the process of receiving information, processing it, and then applying it to make decisions. Tasks that require sustained and voluntary directed attention can be mentally draining.

The urban environment requires greater directed attention that can cause attention fatigue, and the natural environment provides the opportunity to recover from this fatigue with resultant cognitive improvements [142]. The previously cited study by Aspinall *et al.* [139] that employed EEG to measure affect also measured directed attention. While the restorative move from urban to green was found to bring about “a greater range and subtlety of emotional response,” the opposite movement from green to the more attention-demanding urban environment with heavy traffic and more people brought about a clear effect in engagement and alertness and directed attention. In another study, participants were first asked to complete a mentally taxing task. Subsequent video exposure to the natural environment reduced heart rate and subjects performed better on new tasks that demanded directed attention as compared to those who viewed urban settings largely devoid of nature [143]. This heightened cognition brought about by the restorative effects of nature was also experienced in first-hand engagement with the natural environment. Subjects performed better on proofreading tasks after walking in nature as compared to walking in urban environments [138]. Whether viewing images of nature or experiencing it first-hand, exposure to the natural environment is accompanied by restored attention and subsequent improvements in cognition.

We again look to children to understand how the ability of nature to improve attention and cognition might contribute to heightened health. Since exposure to GI can restore attention, it may be able to ameliorate behavioral disorders exacerbated by mental fatigue. One such disorder is attention deficit hyperactivity disorder (ADHD), the most common behavioral disorder among children in the US. The symptoms of ADHD overlap with the symptoms of mental fatigue (e.g., distractability, irritability) caused by sustained and depleted directed attention. When parents were asked about the “aftereffects” of green outdoor activity on their children’s ADHD symptoms, it was found that green outdoor activity had a significantly greater effect than urban outdoor and indoor activities in reducing ADHD symptoms [144].

The ability of exposure to nature to restore attention has also been explored for its role in the self-discipline essential to the performance of many health behaviors. This was tested among girls living in inner-city dwellings [145]. The underlying hypothesis was that if nature can restore attention then it could improve concentration, reduce impulsive behavior, and support the delay of gratification associated with outcomes such as academic achievement, vandalism and violence, and possibly teenage pregnancies. It was found that all three aspects of the girls’ self-discipline (concentration, impulsive behavior, delay of gratification) showed a positive and significant relationship with the greenness of the immediate vicinity that could be viewed from the girls’ homes.

## 11. Social Capital

The transition from the previous section on mental health to social capital is a transition from the *intra*personal to the prominence *inter*personal relations play in an ecological model of health. These interpersonal relations affect one’s social capital or “features of social organization such as networks, norms, and social trust that facilitate coordination and cooperation for mutual benefit” [146]. What has been underappreciated is how the physical environment can facilitate or hinder the social capital that has continually been shown to have a positive influence on both physical and mental health and well-being [147,148].

The importance of social capital on health is evident when it is compared to other behaviors and conditions more commonly associated with an increased risk of mortality. Robert Putnam famously claimed that “poor social capital is as bad as or worse than smoking, obesity, elevated blood pressure, or physical inactivity for human health” [149], and a more recent meta-analysis appears to support this claim. An analysis of 148 studies found a 50% increased likelihood of survival for those with stronger social ties consistent across age, sex, initial health status, and cause of death. Furthermore, social relationships were equally significant as other well established risk factors for mortality [150].

The ability of greenspace to support the various aspects of social capital as defined by Cooper *et al.* [151] has been confirmed by a considerable amount of research into the role of greenspaces in the social ecology of the urban poor in public housing. The results of this body of work confirm the original 19th century beliefs and motivations for introducing parks in cities which posited that greenspace was essential to the social and physical health of urban dwellers, particularly the most disadvantaged. Commenting on GI in the form of urban forestry, Kuo [152] states that disadvantaged urban neighborhoods are

…precisely the context where social ecosystem health is at greatest risk and where urban trees are least present. While poverty is not synonymous with alienation and risk of crime, too many poor urban neighborhoods are characterized by high levels of mistrust, isolation, graffiti, property crime, and violent crime. It may be that the greatest benefits of urban forestry accrue to some of its historically most underserved constituencies. (p. 153)

Years of work by Kuo and her colleagues examining the role of greenspace on the social ecology of the urban poor support this claim.

A telling indicator of whether people are likely to commune in a particular space is their preference for certain types of design features that make spaces more attractive and therefore inviting. Greenery in the form of trees is one such design feature. A photo simulation that compared people’s preferences for communal areas in public housing complexes with varying levels of tree density found that there was a significant preference for communal areas with more trees [153]. While not entirely shocking, and hinting at a biophilic preference [154] for selected representations of nature, more telling and significant to the influence of GI on social capital is that one in three people stated they would use the communal areas more if trees were introduced. Now, preferences and intentions to use a communal space are a good starting point, but do these translate into the actual use of the space necessary for social interactions to occur? It appears so. Observations of these spaces revealed that greener areas attracted larger groups of more heterogeneous ages [155]. In a follow up study, there was 83% more social activity in greenspaces *versus* more barren spaces with the pattern holding across age and gender [156]. Greenspaces were important in supporting social activity across age and gender, but they were particularly important for adults and females. Addressing the possibility that more social activity may simply be a product of a greater total number of people using greenspaces as compared to barren spaces, there was found to be proportionally more social than nonsocial activity in greenspaces when compared to more barren spaces. 

The next step is then to determine if the use of greenspace has a positive effect on social capital. Among residents randomly assigned to 18 architecturally identical public housing facilities, there was found to be a positive association not only between increased levels of greenspace and use of common spaces but also between greenspace and a number of indicators of “neighborhood social ties” (*i.e.*, amount of socializing in the building, familiarity with neighbors, sense of community) [157]. Furthermore, the relationship between greenery and neighborhood social ties was mediated by the use of the common spaces. In other words, more greenery led to more use that resulted in a positive effect on greater social ties in the neighborhood. The relationship between a greener environment and greater social capital also holds true among the elderly who are particularly prone to isolation. Examining the social ties of the elderly in public housing, it was found that those who lived in housing with greater exposure to green common areas had greater involvement with neighborly activities (e.g., talking with neighbors), reported stronger social relationships with friends and neighbors (e.g., greater familiarity with other residents), and had a stronger sense of local community [158].

Greenspaces appear to be important for facilitating social capital as measured in a variety of ways, and it is well established that social capital is a significant determinant of health. One study completes the chain of GI leading to heightened social capital leading to improved health. Looking first at greenspace and health, Maas *et al.* [159] found that people with more greenspace in the immediate neighborhood environment (1km) had better self-perceived health, experienced fewer health complaints in the last 14 days, and had a lower self-rated propensity for psychiatric morbidity. Next, examining greenspace and social capital, they found that more greenspace in one’s environment was associated with fewer feelings of loneliness (at 1 km and 3 km) and less of a perceived shortage of social support (at 1 km). The relationship between greenspace and social support was strongest in the most urban communities and for youth, the elderly, and persons of low socio-economic status, all of which are believed to have lower levels of mobility. Taking these two pieces of information together, they then explored the role of social capital in mediating the relationship between greenspace and health. In testing mediation, or the degree to which loneliness and social support intervened in the relationship between greenspace and health, it was found that loneliness within the immediate and larger environs around one’s home partially mediated self-perceived health, health complaints in the last 14 days, and self-rated propensity for psychiatric morbidity. Shortage of social support in the immediate vicinity partly mediated the relationship between greenspace and health complaints in the last 14 days and more fully mediated the relation between greenspace and self-rated propensity for psychiatric morbidity.

## 12. Conclusions

This survey of the literature has summarized the diverse and complex ways that the presence of, and access and exposure to, GI supports health. The ecosystem services reliant on GI range from those fundamental to human survival (e.g., clean water) to those that enhance health and well-being (e.g., physical activity, mental restoration). Furthermore, most forms of GI simultaneously support myriad ecosystem services and therefore health co-benefits. A GI system that supports health simply by being present in one’s environment (e.g., water quality, climate regulation) can also bring with it co-benefits from the health behaviors that occur when accessing it (e.g., physical activity, social interactions). Green infrastructure as both urban infrastructure and as infrastructure to the biosphere is essential for the continued provision of these health sustaining and promoting ecosystem services.

Infectious disease ecology, physical activity behavior, mental restoration, stormwater management, and social capital are the most developed areas of nature and health research, but, even in these areas, much work needs to be done. Building off the excellent work that has been done and folding in other underrepresented ecosystem services equally fundamental to life and health more accurately reflects the overarching and permeating role of GI in the ecological model of health. We feel that a consistently more accurate accounting of the health co-benefits of GI may spur public health to more fully embrace GI conservation as basic public health practice. There is no greater good that could be done for health promotion than the protection of the GI on which all humans depend.

The purpose of this paper was not to take on the formidable task of identifying the numerous gaps in extant nature and health research. Rather, it was to emphasize that the scope of nature and health research needs to be more consistently comprehensive in its accounting of the array of health-supporting ecosystem services. With this said, there are a number of wonderful references the reader should call on if guidance on research gaps and directions for future research are desired [4,160,161].
